# Do antidepressants change our interpretations of facial emotions?

**DOI:** 10.1017/S0033291726104000

**Published:** 2026-05-06

**Authors:** Nigel McKenzie, Jessica Bone, Larisa Duffy, Marcus Mufano, Glyn Lewis

**Affiliations:** 1 UCL: University College London, UK; 2 University of Bristol, UK

**Keywords:** antidepressants, cognitive neuropsychological model, depression, emotional processing

## Abstract

**Background:**

Cognitive neuropsychological models propose that antidepressants exert their therapeutic effects by modifying negative emotional processing biases early in treatment. However, evidence from large, long-term clinical samples is limited.

**Methods:**

We conducted a mechanistic analysis within the Antidepressants to Prevent Relapse in Depression randomized controlled trial, which compared maintenance antidepressant treatment with placebo substitution in adults with recurrent depression who were currently well (N = 478). Participants completed a computerized facial emotion recognition task at baseline, 12 weeks, and 52 weeks, in which faces morphed from happy to sad. The primary outcome was the number of faces classified as happy (0–45). Linear and longitudinal mixed-effects models were used to compare treatment groups and examine associations with depressive (PHQ-9) and anxiety (GAD-7) symptoms.

**Results:**

Of the 462 participants completing at least one task, there was no evidence that discontinuing antidepressants altered performance compared with maintenance at 12 weeks (adjusted mean difference = 0.23, 95% CI –0.5 to 1.0, p = 0.5) or 52 weeks (0.29, –0.5 to 1.2, p = 0.5). Depressive symptoms were negatively associated with happy face classifications both cross sectionally (β = –0.20 per PHQ-9 point, p = 0.02) and longitudinally (β = –0.09, p = 0.05). Anxiety symptoms were positively associated with happy classifications (β = 0.11, p = 0.047).

**Conclusions:**

Maintenance antidepressant treatment did not sustain positive emotional processing biases as indexed by facial emotion recognition, despite robust associations between such biases and depressive symptoms. These findings challenge the generalizability of laboratory evidence on emotional bias modification to long-term clinical treatment and highlight the need for further mechanistic research on antidepressant action.

## Introduction

Depression is a leading cause of disability worldwide. It was ranked by the World Health Organization as the single largest contributor to global disability in 2015, amounting to 7.5% of all years lived with disability (James et al., 2017; Moussavi et al., 2007). Diagnosing and treating depression is therefore a priority to reduce the global burden of disease. The present main treatment options for depression are antidepressant medication and psychological therapies such as cognitive behavioral therapy (NICE Guidelines, 2022).

The most widely used antidepressants are selective serotonin reuptake inhibitors (SSRIs), which inhibit the reuptake of serotonin into the neuron, thus increasing serotonin transmission. After this initial pharmacological action, there is no consensus about subsequent mechanisms that lead to the improvement in depressive symptoms. It has been proposed that antidepressants work via an effect on the negative self-evaluations, beliefs, and memories that appear to play a key role in depression (Harmer, Duman, & Cowen, 2017; Roiser, Elliott, & Sahakian, 2012). Antidepressant administration increases the relative processing of positive versus negative affective information leading to a reduction of depressive symptoms. These actions should occur relatively quickly following drug administration. To explain the delay seen in treatment response, it has been proposed that the change in affective bias does not directly enhance mood but provides a platform for cognitive and psychological changes that occur over the following weeks. These changes may then lead to the reduction of depressive symptoms (Disner, Beevers, Haigh, & Beck, 2011; Harmer, Goodwin, & Cowen, 2009a). In remitted patients, although overt symptoms have improved, subtle negative processing biases often persist. In contrast, healthy individuals typically show either neutral or mildly positive biases in emotion processing, such as preferential attention to positive stimuli.

Negative biases in information processing that underlie depression have been investigated in various ways. Previous research has looked at mood congruent biases in cognitive tasks (Halahakoon et al., 2020). Tasks include the word recall task that tests memory of socially rewarding and socially critical information (Ahmed et al., 2022; Rodriguez-Sanchez et al., 2023), the “go no-go” decision-making task where participants choose whether to respond to stimuli and receive rewards for responding to certain stimuli and punishments for responding to others (Malamud et al., 2024) and the facial emotion processing task (referred to as the face task in the following). One version of the face task involved asking participants to identify six emotions: whether the facial expression presented was displaying happiness, sadness, fear, anger, disgust, or surprise. For each emotion, the faces morphed in 15 steps from displaying no emotion to displaying the emotion in full intensity. Using this version of the face task with a cohort of patients attending GP practices, some evidence has been found for an association between depressive symptoms and recognition of happy and sad faces (Bone et al., 2019). Other versions of facial emotional recognition tasks have been used to investigate mood congruent biases (Dam et al., 2021; Monferrer et al., 2023; Van Vleet et al., 2019). Laboratory studies in healthy volunteers have shown that short-term administration of SSRIs can shift processing toward positive emotional stimuli without altering subjective mood (Harmer, O’Sullivan, et al., 2009b; Harmer, Shelley, Cowen, & Goodwin, 2004). However, evidence from large clinical populations is scarce, and it remains unclear whether these effects persist during long-term treatment, in patients who have recovered from depression, and whether they mediate protection against relapse.

This study investigates the effect of antidepressants on the recognition of mood in faces displaying varying intensities of happiness or sadness. We used a simplified version of the face task used in previous studies, with faces only displaying happy and sad emotions. It is anticipated that by using a face task focusing on just the two emotions of interest, the spectrum from very sad to very happy, it will be possible to determine with greater precision whether antidepressants affect the recognition facial expressions as happy or sad. The intervention was discontinuing antidepressants for participants who were previously being treated with antidepressants. It was hypothesized that this would lead to a reduction in monoamine levels resulting in an increased bias toward negative information processing in the intervention group, with fewer faces rated as happy.

A secondary objective of this study was to investigate whether symptoms of depression and anxiety affect performance on the face task. Previous studies have found some evidence for a negative association between depression and the threshold for recognizing a face as showing a happy emotion (Bone et al., 2019). Fewer studies have investigated the effect of anxiety symptoms on the face task and previous evidence on how anxiety symptoms affect performance on the face task is less clear cut (Attwood et al., 2017; Berg et al., 2016; Dyer, Attwood, Penton-Voak, & Munafò, 2022). The conclusion from one of these studies was that there was some evidence for reduced accuracy in recognition of facial emotions with increased anxiety (Dyer et al., 2022).

## Methods

### Study design and participants

Data for this article come from the Antidepressants to Prevent Relapse in Depression (ANTLER) trial (Duffy et al., 2019; Lewis et al., 2021). This was a double-blind randomized controlled trial (RCT) with a primary aim to determine whether people who have recovered from an episode of depression and are maintained on antidepressants will be less likely to experience a relapse, compared with people whose medication has been switched to placebo. Participants were aged between 18 and 74 and had reported at least two previous episodes of depression and had been taking antidepressants (citalopram, sertraline, fluoxetine, or mirtazapine) for more than 2 years. All participants were taking antidepressants at baseline but were well enough to consider stopping their medication. The main exclusion criterion was current depression. In total, 478 patients from 150 general practices were recruited into the trial.

Participants were randomized to a maintenance group that continued antidepressant treatment or a discontinuation group. Those in the discontinuation group had their antidepressant medication gradually reduced so that after 8 weeks they were receiving only placebo medication (the regime of fluoxetine reduction differed slightly from the other three antidepressants due to its longer half-life). Randomization was stratified by site (Bristol, London, Southampton, and York), antidepressant medication and severity of depressive symptoms.

Participants recruited into the trial were assessed at baseline, when all participants were taking antidepressant medication, and at 6, 12, 26, 39, and 52 weeks from randomization. Demographic data were collected at baseline.

The ANTLER trial main analysis found that the time to relapse of depression was shorter in the discontinuation group, with a hazard ratio of 2.06 (95% CI 1.56–2.70; p < 0.001). Over the following year, relapse was experienced by 39% of those who continued antidepressants and 56% of those who discontinued antidepressants (Lewis et al., 2021).

A subsidiary objective of the ANTLER trial was to investigate the mechanistic outcomes of discontinuing antidepressants. The trial protocol included three computerized emotional processing tasks to investigate the neuropsychological markers of antidepressant action (Duffy et al., 2019). These were the word recall task (analyzed previously) (Rodriguez-Sanchez et al., 2023), the “go no-go” decision-making task and the face task.

### Measures

#### Depression and anxiety

At each follow-up, the retrospective Clinical Interview Schedule-Revised (20) scale was used to assess whether there had been a relapse of depression. Participants were also evaluated on eight secondary outcomes including depressive symptoms using the Patient Health Questionnaire 9-item version (PHQ-9) (Kroenke, Spitzer, & Williams, 2001) and anxiety symptoms using the Generalized Anxiety Disorder Assessment 7-item version (GAD-7) (Spitzer, Kroenke, Williams, & Löwe, 2006).

#### The face task

**Figure 1.**
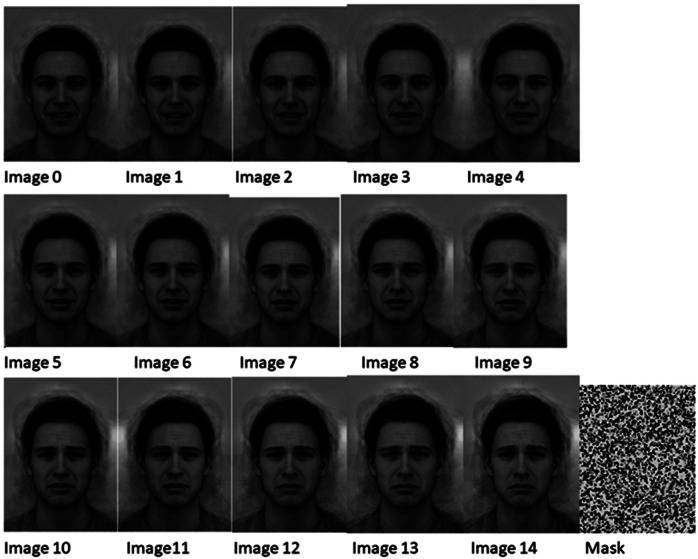
Face task images.

The ANTLER face task was administered to participants at baseline and at 12 and 52 weeks, using a program on a laptop computer. Participants were shown the 15 images in Figure 1 in random order with the mask image shown between the face images. Faces morph incrementally in 15 steps from very happy to very sad, with image 8 as the neutral face. Each face was shown for 500 ms followed by the mask image for 250 ms. Participants were instructed to press key “c” on the laptop keyboard if they thought the face was happy and key “m” if they thought the face was sad. The program was designed so that the next face was not shown until either key had been pressed. The sequence of 15 faces was then shown in random order two more times, making a total of 45 faces shown to participants. The task took about 5 min to complete.

Task performance was measured using the number of faces rated happy, which could range from 0 to 45. A higher score indicates that the participant is rating less extreme expressions as happy, and correspondingly, not as sad (given the decision required is either sad or happy).

### Data analysis

Analyses were performed using Stata 17. Descriptive statistics are presented separately for each treatment group. In regards to participants being noncompliant with the initially prescribed medication, the trial protocol indicated that an intention to treat analysis of trial outcome data would be made. An intention to treat approach was adopted for the data analysis in this article.

For the primary analysis, an initial linear regression was performed separately for the two follow-up time points. The model had face task score (number of faces rated happy) for each participant at each time point as the outcome variable, and treatment group as predictor variable, with face task score modeled as a continuous variable. In addition, face task score at baseline and the minimization variables used in the randomization procedure (site, antidepressant medication, depressive symptom severity), were included in the model as covariates to allow for any chance imbalances following randomization.

To take account of missing data a model proposed by Liang and Zeger Scott (2000) was used. This is a modification to the longitudinal linear regression model, which enables missing data to be included in the model. The model includes the baseline value of the outcome variable as an outcome rather than a predictor variable. The mean value of the outcome variable at time zero is constrained to be the same for both treatment groups due to randomization. Liu et al. (2009) showed that, when there is no missing data, this model again produces the same parameter estimates as the regression model at separate time points although the variance estimates are slightly different. Liu et al. also showed that, when the data are missing at random, the constrained longitudinal regression model yields parameter estimates that are asymptotically unbiased when the data are from multivariate normal distributions.

As noted in the Section “Introduction,” a secondary aim of this study was to investigate whether there is an association between the number of faces rated as happy and symptoms of depression or anxiety using data from the PHQ-9 and GAD-7 scales collected in the trial. As already noted, previous studies have found some limited evidence for an association between performance on the face task as outcome, and depression and anxiety. The following describes the methods used for this secondary analysis.

Using collected ANTLER trial data as an observational dataset, an initial exploratory regression analysis was performed with face task score (the number of faces rated happy) as the outcome variable, and PHQ-9 and GAD-7 both included as explanatory variables. This indicated age was a strong confounder and age was therefore included in the regression model.

A sensitivity analysis of missing data for face task score was performed using a logistic regression with missingness as the outcome. Demographic and minimization variables as well as group and time were included in the model (see results in Supplementary Table S2 in the Supplementary Appendix). Group was significantly associated with missingness with the discontinuation group having approximately twice the odds of the maintenance group for data to be missing. Female participants were less likely to have missing count data compared to males and some sites were more likely to have missing data than London. Age and employment status were not associated with missingness. Based on the sensitivity analysis and after taking into account the clinical factors underlying the ANTLER trial, the randomized nature of the trial, and the secondary objective of participants being asked to perform the face task, it was deemed appropriate to assume the outcome was missing at random in the analyses, after adjusting for variables associated with missingness.

In order to take account of the data collected at all time points, a mixed-effects random intercept model for repeated measures was used which allows for differences between individual variances on repeated performance of the face task. The fixed effects explanatory variables were again selected to be PHQ-9, GAD-7, and age based on the earlier exploratory analyses. The mixed model includes 1219 observations from 461 participants (data were missing for one participant for PHQ-9 and GAD-7 hence only 461 of the 462 participants who performed the face task were included). This model makes better use of the available data than the previous regression model. The model was run again adjusted for variables associated with missingness (group, sex, site, and medication).

## Results

Of the 478 participants in the ANTLER trial, 462 completed the face task at least once. At baseline, when all participants were still taking antidepressants, 228 in the maintenance group, and 223 in the discontinuation group (total 451) completed the face task. The number of faces rated happy on the face task at baseline was similar for both groups, centered around a mean of 18 (SD = 4.8) and approximately normally distributed (see Supplementary Figure S1 in Supplementary Appendix).

The total number of participants with data on the face task at each time point reduced from 451 at baseline (98% of the total sample, n = 462), to 411 at 12 weeks (89%), and 358 at 52 weeks (77%). In the maintenance group, 184 participants completed the task at every time point compared to 155 in the discontinuation group. There was no missing data for baseline demographic or minimization variables.

### Adherence to trial medication

Adherence to trial medication among the 462 participants who completed at least one face task reduced from 100% at baseline to 86% overall at 12 weeks and 50% overall at 52 weeks. In the maintenance group, 136 (59%) participants, who remained on trial medication, completed the face task at all three time points compared with 72 (31%) in the discontinuation group. Reasons for changes in participants’ medication included relapse or, if following a participant review by his/her GP, a medication change was thought to be appropriate.

Depressive symptoms measured by PHQ-9 were the same for both groups at baseline and 52 weeks but depressive symptoms were higher in the discontinuation group at 12 weeks: maintenance group 4.0, discontinuation group 6.4, (p < 0.001). More details of outcomes at all time points are available in the ANTLER trial paper.

### Demographics

Table 1 shows the demographic data for the 462 participants who completed at least one face task. More females than males took part in the trial with similar percentages in both groups. The age range was wide from 19 to 74 years but with a distribution skewed toward older ages. Employment status was similar in both groups with 55% and 64% employed in maintenance and discontinuation groups, respectively. Approximately twice as many participants came from London compared to the other three sites.

**Table 1.** Demographic variables at baseline

	Maintenance group (*N* = 232)	Discontinuation group (*N* = 230)
Age in years (median [IQR])	55 [46–64]	55 [48–64]
Female sex	165 (71%)	176 (77%)
Employment status		
Employed	135 (58%)	147 (64%)
Retired	71 (31%)	64 (28%)
Other	26 (11%)	19 (8%)
Trial site		
London	100 (43%)	95 (41%)
Bristol	48 (21%)	54 (23%)
Southampton	48 (21%)	48 (21%)
York	36 (16%)	33 (14%)

### Association of face task score with treatment group at each follow-up point

Due to increasing numbers of participants no longer compliant with medication at successive follow-up time points, an initial regression analysis was performed separately for the two follow-up times. These regression models restrict the numbers included in the analysis to those participants who completed the face task both at baseline and at the relevant follow-up time points.

At both 12- and 52-week follow-ups, the discontinuation group had a slightly higher estimated mean score on the face task compared to the maintenance group after adjustment for baseline face task score and minimization variables. However, these differences were not significant, as shown in Table 2. Thus, there was no evidence that discontinuing treatment with antidepressants altered performance on the face task.

**Table 2.** Face Task Outcome scores means and differences, with 95% confidence intervals, by group at different time points

Group	Maintenance	Discontinuation	Difference
Regression model at fixed times	Mean	Mean	Coefficient [95% confidence interval]
12 weeks (400 obs)	18.4 [17.8, 18.9]	18.6 [18.0, 19.1]	0.21 [0.5, 1.0] p = 0.6
52 weeks (353 obs)	19.5 [18.9, 20.1]	19.9 [19.2, 20.5]	0.37 [0.5, 1.3] p = 0.4
Constrained longitudinal regression model (allows for missing data)			
12 weeks (462 groups 1220 obs)	18.4 [17.9, 19.0]	18.7 [18.1, 19.2]	0.23 [0.5, 1.0] p = 0.5
52 weeks (462 groups 1220 obs)	19.5 [18.9, 20.1]	19.8 [19.1, 20.4]	0.29 [0.5, 1.2] p = 0.5
Constrained longitudinal regression model for patients who remained on allocated medication			
12 weeks (231 groups 667 obs)	18.8 [18.1, 19.5]	18.7 [17.8, 19.7]	0.03 [1.1, 1.0] p = 0.9
52 weeks (231 groups 667 obs)	19.6 [18.7, 20.3]	19.9 [18.9, 20.8]	0.33 [0.8, 1.5] p = 0.6

As would be expected, given that the face task was completed at baseline and at follow-up times by each participant, performance at baseline was strongly related to performance at both follow-ups.

### Constrained longitudinal regression analysis

The constrained longitudinal regression analysis allows for missing data for all participants who completed at least one face task (n = 462) to be included in the analysis, regardless of whether they have missing data. A separate longitudinal regression analysis was completed for participants who completed at least one face task and adhered to treatment allocation. The results are summarized in Table 2.

This model again shows only a small difference in mean count scores between the two treatment groups at the two time points after adjusting for other variables. The large p-values again provide no evidence of a difference in scores on the face task between the maintenance and discontinuation groups.

Thus, in regards to the primary analysis, we found no evidence of a difference in mean count scores on the face task between participants taking antidepressants and those taking placebo at 12- and 52-week follow-ups.

### Is performance on the face task associated with depression and anxiety?

As noted above, a secondary aim of the study was to investigate whether there was an association between the number of faces rated as happy and symptoms of depression or anxiety. The results using only baseline data are shown in Table 3. The results show evidence for a negative association between the number of faces rated as happy and depressive symptoms as measured by the PHQ-9 scale. The results indicate 0.2 fewer faces were rated happy for each unit increase in the PHQ-9 score (p = 0.02). There was also weak evidence for a positive association between the number of faces rated happy and generalized anxiety symptoms as measured by the GAD-7 scale. The number of faces rated happy increased by 0.2 for each unit increase in GAD-7 (p = 0.06 or p = 0.08 after adjusting for missingness).

**Table 3.** Associations between performance on the face task, depressive symptoms (measured using the PHQ-9), and anxiety symptoms (measured using the GAD-7)

Linear regression model at baseline: 450 observations^a^		
Variable	Parameter estimates	Adjusted for missingness
PHQ-9	−0.20 [−0.37, −0.03] p = 0.022	−0.20 [−0.37, −0.03] p = 0.021
GAD-7	0.19 [−0.01, 0.39] p = 0.061	0.18 [−0.02, 0.38] p = 0.081

Longitudinal mixed-effects model: 1219 observations 461 groups		
Variable	parameter estimates	adjusted for missingness
PHQ-9	−0.09 [−0.19, −0.001] p = 0.052	−0.09 [−0.19, 0.001] p = 0.054
GAD-7	0.11 [−0.01, 0.21] p = 0.041	0.11 [0.001, 0.21] p = 0.047

a PHQ-9 data were missing for one participant at baseline.

Based on the analysis including data from all timepoints (baseline, 12 weeks, and 52 weeks), there was again evidence for a negative, although smaller, association between the number of faces rated happy and depressive symptoms. There was, again, a positive association between number of faces rated happy and generalized anxiety symptoms.

## Discussion

In this large randomized trial, we found no evidence that discontinuing maintenance antidepressant treatment altered recognition of happy versus sad facial expressions compared to continuing treatment at either 12 or 52 weeks. However, we observed consistent associations between depressive symptom severity and reduced identification of happy faces, and a positive association between anxiety symptoms and happy ratings.

Similar results have been found of no difference between those maintained on antidepressants and those in the discontinuation group, on performance of the word recall task (Rodriguez-Sanchez et al., 2023) and the go no-go task (manuscript in preparation).

Our findings contribute to ongoing debates regarding the cognitive neuropsychological mechanisms of antidepressants. The absence of an effect on facial emotion recognition challenges the assumption that long-term maintenance of antidepressants exerts its therapeutic benefit via sustained normalization of emotional processing biases. This contrasts with earlier small-scale studies in healthy volunteers showing early changes in emotional bias following short-term antidepressant administration (Harmer et al., 2004, 2009a). Our results suggest that such early laboratory findings may not generalize to long-term treatment in clinical populations, or that the specific task employed here may not capture the relevant cognitive processes.

Previous research has indicated some evidence for an association between score on the face task and depressive symptoms (Bone et al., 2019). People who are depressed are more sensitive to negative affect and show a negativity bias when interpreting ambiguous stimuli. The results from this study provide stronger evidence in support of these previous findings, namely that the threshold for seeing a morphed face as happy may increase with increased depressive symptoms.

The evidence that an increase in anxiety symptoms was associated with a higher frequency of rating faces as happy for participants with the same age and depression levels was surprising. A possible explanation for this finding is that some degree of anxiety can improve performance on difficult tasks, for example the anxiety experienced by musicians and actors immediately before a performance (Yerkes–Dodson law) (Yerkes & Dodson, 1908). Recognizing the more ambiguous faces as happy, in the very short time given to participants who performed the face task, was a difficult task. Then, 7 out of the 15 faces were designed to display some degree of happiness so a mean score of 21 on the face task would correspond with a fully correct identification of happy faces, whereas the mean score on the face task for all participants at baseline was 18. It seems plausible, therefore, that some level of anxiety could have resulted in improved performance on the task.

### Strengths and weaknesses

A strength of the study is that the data come from an RCT and so this will have eliminated confounding for the analysis of the antidepressant effect on the face recognition task. However, over time there was a departure from the medication allocated to participants at randomization. At 12-week follow-up, 86% of participants were compliant with their allocated treatment but only 50% were compliant at 52-week follow-up. Clearly, the primary aim of their care is to keep the participants, who have a history of depressive illness, as symptom free as possible. Those who reported a worsening of symptoms during follow-up had their medication reviewed, with changes made as considered appropriate by their GP. Nevertheless, results from 12-week follow-up should provide a reliable test of the effects of antidepressants on how participants perceived the mood of the faces shown in the face task. Adjustment was made for available predictors of missingness, in terms of participants who did not complete the face task at all time points, but this did not alter the results.

The number of participants selected for the ANTLER trial was based on a requirement to detect a 1.92 hazard ratio for relapse in the discontinuation group compared to the maintenance group based on current estimates of known relapse rates. It is possible that a trial with larger numbers might have detected a difference between performance on the face task between the groups although the numbers included in this study are much larger than those included in previous investigations of this question.

### Implications

These results have important implications in that they do not support the proposed cognitive theories (Disner et al., 2011) for the mechanisms of action of SSRIs. Similar negative findings have been published on the go/no-go decision-making task and the word recall task using data from the ANTLER and PANDA trials (Ahmed et al., 2022; Malamud et al., 2024; Rodriguez-Sanchez et al., 2023). There was no evidence of a difference on performance of these three emotional processing tasks by participants who were taking maintenance antidepressant medication compared to those who discontinued their medication and were taking a placebo. This contrasts with previous research (Harmer, O’Sullivan, et al., 2009b) that had found some evidence that short-term administration of antidepressants had an effect on emotion-related tasks in healthy volunteers, reducing the psychological processing of negative relative to positive emotional material. A possible reason why this study does not confirm the results from these earlier smaller experimental studies could be that this study looks at effects over a longer time period and the effects of antidepressants on cognitive mechanisms might be only short term. It could also be that the face task used in this study is not linked to the cognitive mechanisms that are affected by antidepressants. This study only addresses negative biases to facial stimuli and not to verbal stimuli or other forms of visual stimuli. Also this study investigates changes linked to discontinuation of medication in remitted patients rather than the effect of initiation of treatment in depressed patients. Previous findings were based on small samples and this increases the risk of false positive results (Button et al., 2013).

These findings highlight the need for further research into the cognitive mechanisms that underlie depression. If further advances can be made in understanding the cognitive mechanisms that contribute to depression this should improve the prospects for the discovery of new, more effective antidepressants.

## Supporting information

10.1017/S0033291726104000.sm001 McKenzie et al. supplementary material McKenzie et al. supplementary material

## Supplementary material

The supplementary material for this article can be found at http://doi.org/10.1017/S0033291726104000.
