# Supplementary material for: Do antidepressants change our interpretations of facial emotions?
Source: Psychol Med. 2026 May 6;56:e132. doi: 10.1017/S0033291726104000 (PMC13161807; doi:10.1017/S0033291726104000)
Supplement: McKenzie et al. supplementary material [file S0033291726104000sup001.docx]

**Supplementary Appendix**

The face task with outcome count, the number of faces rated happy, was performed by 451 participants at baseline. The distribution of count scores at baseline is shown in Figure S1. The scores appear to be approximately normally distributed.

**Figure S1** Distribution of face task count scores at baseline
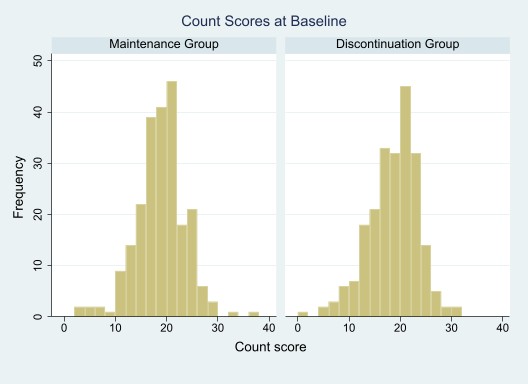


Table S1 shows the mean count score on the face task for each treatment group, and the estimated difference between group means, at baseline and each follow-up time.

**Table S1** Count scores on face task at different time points

|  | Baseline (mean, SD, n) | 12 weeks | 52 weeks |
| --- | --- | --- | --- |
| Maintenance grp | 18.4 (4.8, 228) | 18.5 (4.4, 212) | 19.5 (4.4, 195) |
| Discontinuation grp | 18.1 (4.8, 223) | 18.5 (4.8, 199) | 19.7 (4.3, 163) |
| Difference | 0.25 95%CI [-0.65,1.14] | 0.08 95%CI [-0.82, 0.97] | -0.18 95%CI [-1.09, 0.73] |

There was missing data for the face task at baseline, 12 weeks and 52 weeks among the 478 participants in the ANTLER trial. A logistic regression was performed with missingness as the outcome to identify variables associated with missingness. Data from baseline, 12 weeks and 52 weeks were combined. Demographic and minimisation variables were included in the model. Results of logistic regression with missingness as outcome variable are shown in Table S2

| **Table S2** Odds ratios for missing data using baseline variables as predictors of missingness | | |
| --- | --- | --- |
| predictor variables | odds ratio | p |
| group | 0.48 [0.35, 0.67] | 0.00 |
| age at randomisation | 1.00 [0.98, 1.02] | 0.91 |
| sex male v female | 1.61 [1.14, 2.26] | 0.01 |
| employed |  |  |
| retired | 1.04 [0.66, 1.63] | 0.88 |
| other | 1.12 [0.64, 1.97] | 0.70 |
| site: London |  |  |
| Bristol | 0.57 [0.38, 0.88] | 0.01 |
| Southampton | 0.89 [0.55, 1.42] | 0.62 |
| York | 0.21 [0.14, 0.32] | 0.00 |
| medication: sertraline |  |  |
| citalopram | 1.46 [0.94, 2.24] | 0.09 |
| fluoxetine | 1.44 [0.91, 2.27] | 0.12 |
| mirtazapine | 3.04 [1.07, 8.59] | 0.04 |
